# Patient-Reported Outcomes After Arthroscopic Single-Row Rotator Cuff Repair: A Monocentric Retrospective Study at Minimum 12 Years Follow-Up

**DOI:** 10.3390/jcm14010235

**Published:** 2025-01-03

**Authors:** Alessandro Castagna, Tommaso Campeggi, Marco Maria Minelli, Mario Borroni, Marco Conti, Giacomo Delle Rose, Raffaele Garofalo, Riccardo Ranieri

**Affiliations:** 1 Department of Biomedical Sciences, Humanitas University, Via Rita Levi Montalcini 4, 20072 Pieve Emanuele, Italy; acastagna@me.com (A.C.); marcomariaminelli@gmail.com (M.M.M.); riccardo.ranieri@humanitas.it (R.R.); 2 IRCCS Humanitas Research Hospital, Via Manzoni 56, 20089 Rozzano, Italy; mario.borroni@gmail.com (M.B.); conti.marco@gmail.com (M.C.); giacomo.delle_rose@humanitas.it (G.D.R.); raffaelegarofalo@gmail.com (R.G.); 3 Shoulder and Sport Medicine Unit, Miulli Hospital, Strada Prov. 127 Acquaviva—Santeramo Km, 4, 70021 Acquaviva delle Fonti, Italy

**Keywords:** shoulder, rotator cuff, arthroscopy, double row, single row, retear rate, patient satisfaction, shoulder pain, PROMS

## Abstract

**Background:** Arthroscopic rotator cuff repair (RCR) is a common procedure, yet long-term patient-centered outcome studies remain limited. This study aims to evaluate the outcomes of arthroscopic RCR using a single-row metallic anchor technique over a 12-year follow-up, focusing on patient-reported outcomes and potential risk factors. **Methods:** A monocentric cohort study was conducted on patients who underwent complete arthroscopic RCR with a single-row metallic anchor technique from January 2007 to July 2011. A total of 628 patients completed three standardized questionnaires: Quick Disabilities of the Arm, Shoulder and Hand (QuickDASH), American Shoulder and Elbow Surgeons Standardized Shoulder Assessment Form (ASES), and the Simple Shoulder Test (SST). They were also asked for their overall satisfaction on the procedure with a single direct question. Outcomes were analyzed by gender, age at surgery, lesion pattern, and follow-up duration. **Results:** The mean follow-up period was 12.9 ± 1.3 years. Overall satisfaction was 96.5%. The mean scores for QuickDASH, ASES, and SST were 7.2 ± 15.7, 83.8 ± 23.9, and 89.9 ± 22.3, respectively. Female patients reported significantly lower QuickDASH (*p* < 0.001), ASES (*p* < 0.001), and SST (*p* = 0.004) than male patients, but overall satisfaction did not differ by gender. Age, number of tendons repaired, anterior tear, and follow-up length were not significantly associated with differences in outcome measures. **Conclusions:** Single-row arthroscopic RCR provides excellent long-term patient-reported outcomes and high patient satisfaction. Female gender may be associated with slightly lower functional scores, yet satisfaction remains unaffected. This study supports the single-row technique as a reliable, efficient, and cost-effective option for long-term success in RCR, challenging the need for more complex multi-anchor approaches.

## 1. Introduction

Rotator cuff disorders are the most common cause of disability related to the shoulder [1]. In particular, rotator cuff tears (RCTs) affect approximately 25% of individuals in their 60s and 50% of individuals in their 80s, resulting in pain and functional limitation [1]. This pathological impact on patients’ quality of life was demonstrated to be comparable to that of congestive heart failure, diabetes, myocardial infarction, and depression [2]. Due to the last decade’s continuous development in diagnostic and surgical techniques, RCTs’ diagnosis and related procedures have increased exponentially. Based on projection models, costs sustained by the Italian national health care system for RCT management are expected to be over 1 billion euros in 2025 [3]. Arthroscopic rotator cuff repair (RCR) is expected to provide symptom relief, better functioning, and a higher quality of life. However, whether subjective outcomes persist over time is still controversial: large-scale studies on long-term arthroscopic RCR outcomes are few and variations in indications and treatment exist in different health care systems [4]. In this scenario, long-term follow-up studies for rotator cuff repair are needed to prompt patient-centered perspectives. Since the last decade of the 20th century, validated patient-oriented measures have been developed to match the gap created by objective assessment, adding a new dimension to clinical outcome evaluation. Patient-reported outcome measures (PROMs) collect subjective information reported by the patients themselves without interpretation by other parties. These include patient satisfaction and subjective evaluations on symptoms, functioning, and health-related quality of life [5]. Many PROMs have been developed and validated for shoulder pathologies, such as the ASES (American Shoulder and Elbow Society Score), QuickDASH (Quick Disability of the Shoulder, Elbow and Hand score), SPADI (Shoulder Pain and Disability Index), and the SST (Simple Shoulder Test) [6]. The primary aim of this study was to examine long-term patient-reported outcomes of arthroscopic rotator cuff repair with a single-row metallic suture anchor technique. The secondary aim of the study was to analyze whether gender, age at surgery, the number of torn tendons, and length of follow-up could be considered risk factors for worse outcomes after arthroscopic RCR.

## 2. Methods

This is a monocentric retrospective study on a consecutive series of patients who underwent arthroscopic RCR with a single-row suture metallic anchor technique from January 2007 to July 2011 and were contacted at a minimum follow-up of 12 years. Inclusion criteria encompassed patients older than 16 at surgery, who underwent arthroscopic complete RCR with a single-row suture metallic anchor in the same center, with patients able to understand and communicate in Italian, and where minimum follow-up had to be more than 12 years. Exclusion criteria encompassed patients younger than 16 years old, patients who underwent arthroscopic RCR with techniques other than the single-row suture metallic anchor, patients who underwent partial rotator cuff repair, patients who underwent shoulder arthroscopic surgery for conditions other than RCTs, patients unable to understand and communicate in Italian, and bedridden, paralyzed or deceased patients.

### 2.1. Clinical Outcomes

Three distinct standardized and validated questionnaires (QuickDASH, ASES and SST) were administered to the selected patients through a telephonic interview. QuickDASH results were considered *excellent* if the total score was ≤5 points; *good* if the score was 6–15 points; *satisfactory* if the score was 16–35; or *poor* if the score was >35 [7]. ASES results were considered *excellent* if the total score was ≥90 points; *good* if the score was 75–89 points; *satisfactory* if the score was 51–74; or *poor* if the score was ≤50 [8,9].

In addition, patients were able to express their general satisfaction by answering whether they were satisfied with the treatment received and they would have had the surgery repeated, or if they would have preferred a different approach to the disease. The interviewer was a third-party person, a resident doctor not involved in the surgical procedures. All questionnaires were submitted by a single interviewer, who was available for clarifying and explaining question meaning and reasons whenever necessary.

### 2.2. Population

Eligible subjects included 1000 consecutive patients who underwent arthroscopic single-row metallic suture anchor RCR in a timeframe starting in January 2007 and ending in July 2011. Among these, 372 subjects did not meet inclusion criteria, and were eventually excluded: 32 subjects were deceased, 12 bedridden or paralyzed, 156 unwilling to answer, and 172 with an inexistent, wrong, or absent telephone number. The remaining 628 patients (follow-up rate 62.8%) successfully completed the questionnaires. Demographic characteristics of the population included are presented in Table 1.

### 2.3. Statistical Analysis

Categorical data were summarized using numerical counts and percentages. Continuous data were presented in the form of either mean or median values, along with standard deviations or ranges, all accompanied by 95% confidence intervals. In addition, the percentage of patients with an excellent score (for ASES, QuickDASH) and a perfect score (100 for SST) was also calculated.

For risk factor analysis, patients were stratified according to gender, age at surgery (<50 years, 50–59 years, 60–69 years, and ≥70 years), number of torn and repaired tendons (1, 2, and >2), and an anterior extension of the tear (as subscapularis repair required). The association between categorical data was assessed with the chi-square or Fisher’s exact test, as appropriate. The normality of data was assessed with the Shapiro–Wilk test. A t-test or Mann–Whitney U test was used to explore differences for continuous variables, as appropriate. Continuous variables were analyzed to find a correlation through the Pearson and Spearman Rank test. If a significant correlation was found between an outcome measure and a risk factor, the probability of having an excellent or perfect score was calculated with the odds ratio and reported with 95% confidence intervals and the *p* value.

A multivariate linear regression was performed to assess the relation between QuickDASH, ASES, SST and the explanatory variables: gender, age at surgery, number of tendons repaired (1, 2, 3) and presence of anterior tear. Data were checked for multicollinearity with the Belsley–Kuh–Welsch technique. Heteroskedasticity and normality of residuals were assessed, respectively, by the White test and the Shapiro–Wilk test.

Results were statistically significant if the *p* value was <0.05.

Statistical analysis was performed with EasyMedStat (version 3.37.1; www.easymedstat.com, accessed on 24 October 2024).

## 3. Results

### 3.1. Overall Results

At follow-up, the mean QuickDASH score was 7.2 ± 15,7 (95% CI = 6.0–8.4), the mean ASES score was 83.8 ± 23.9 (95% CI = 81.9–85.7), and the mean SST was 89.9 ± 22.3 (95% CI = 88.1–91.6). The median values (with relative Q1–Q3) for QuickDASH, ASES, and SST were, respectively, 0 (Q1 = 0—Q3 = 4.5), 100 (Q1 = 61.7—Q3 = 100), and 100 (Q1 = 91.7—Q3 = 100). The percentage of patients with an excellent score was 76.3% for QuickDASH and 69.7% for ASES; 462 (73.6%) patients had a perfect SST. The overall satisfaction question was answered positively by 606 (96.5%) patients.

### 3.2. Results and Risk Factors (Table 2)

#### 3.2.1. Sex

Woman had a significantly lower QuickDASH (4.81 ± 12.1 vs. 9.9 ± 18.5 *p* < 0.001), ASES (87.2 ± 21.6 vs. 79.9 ± 25.7 *p* < 0.001), and SST (92.9 ± 18.3 vs. 86.4 ± 25.8 *p* = 0.004) compared to men, even if the satisfaction rate did not differ significantly between the two groups.

Male patients compared to females had a significantly higher proportion of excellent scores for QuickDASH (82.8% vs. 68.9%; OR = 0.46; CI [0.32; 0.67]; *p* < 0.001; *p* < 0.001), ASES (75.9.0% vs. 62.8%; OR = 0.54; CI [0.38; 0.76]; *p* < 0.001), and perfect scores for SST (77.4% vs. 69.3%; OR = 0.66; CI [0.46; 0.94]; *p* = 0.026) (Table 2).

#### 3.2.2. Age at Surgery

No significant differences were found among the different groups of age for any scores and for satisfaction rate (Table 2).

#### 3.2.3. Tendon Repair Pattern

No significant differences in outcome scores or satisfaction rate were found considering the number of tendons repaired or the presence of an anterior tear (Table 2).

#### 3.2.4. Length of Follow-Up

No significant correlations were found between length of follow-up and any scores or satisfaction rate (Table 2).

**Table 2:** Results according to sex, age, and tendon pattern for univariate analysis. Values are expressed as mean ± standard deviation.

Parameters	QuickDASH		ASES		SST		Satisfaction	
Sex								
M	4.81 ± 12.1	*p* < 0.001	87.2 ± 21.6	*p* < 0.001	92.9 ± 18.3	*p* = 0.004	322 (97.0%)	*p* = 0.623
F	9.9 ± 18.5		79.9 ± 25.7		86.4 ± 25.8		284 (96.0%)	
Age								
<50 years old	6.9 ± 18.0	0.302	85.3 ± 24.0	0.389	91.3 ± 22.4	0.287	119 (95.2%)	0.351
51–59	8.3 ± 16.5		82.1 ± 25.3		88.5 ± 24.4		229 (97.9%)	
60–69	6.1 ± 13.7		83.9 ± 23.3		91.3 ± 20.0		207 (96.3%)	
≥70	7.3 ± 13.7		86.8 ± 19.2		86.9 ± 21.8		51 (94.4%)	
N^ Tendons Repaired								
Single tendon	6.5 ± 14.4	0.531	84.2 ± 23.6	0.309	91.2 ± 20.7	0.14	414 (95.6%)	0.199
2 tendons	9.1 ± 19.3		82.1 ± 25.7		86.3 ± 26.7		132 (98.5%)	
>2 tendons	7.9 ± 15.0		84.4 ± 22.0		88.3 ± 22.5		60 (98.4%)	
Anterior Tear								
Yes	8.3 ± 17.4	0.242	83.1 ± 24.3	0.352	88.9 ± 22.6	0.515	146 (98.0%)	0.317
No	6.9 ± 15.1		84.0 ± 23.8		90.2 ± 22.3		460 (96.0%)	

#### 3.2.5. Results According to Multivariate Analysis (Figure 1)

In multivariate analysis, the female sex was associated with higher values of QuickDASH (β = 5.36, [2.82; 7.91], *p* < 0.0001). Age at surgery (β = −0.09, [−0.23; 0.04], *p* = 0.1843), anterior tear (β = 0.08, [−4.74; 4.89], *p* = 0.9744), a number of tendons = 3 (β = 1.81, [−4.25; 7.86], *p* = 0.558), and number of tendons = 2 (β = 3.08, [−1.17; 7.34], *p* = 0.1554) were not associated with QuickDASH.

The female sex was associated with lower values of ASES (β = −7.62, [−11.36; −3.87], *p* < 0.0001). A number of tendons = 2 (β = −2.03, [−7.99; 3.92], *p* = 0.5025), anterior tear (β = −1.31, [−8.29; 5.68], *p* = 0.713), age at surgery (β = 0.08, [−0.12; 0.28], *p* = 0.4267), and number of tendons = 3 (β = 1.12, [−8.27; 10.52], *p* = 0.8147) were not associated with ASES.

A number of tendons = 2 (β = −7.73, [−14.19; −1.26], *p* = 0.0193) and the female sex (β = −6.68, [−10.33; −3.03], *p* = 0.0003) were associated with lower values of SST. A number of tendons = 3 (β = −7.3, [−16.22; 1.61], *p* = 0.1083), age at surgery (β = 0.07, [−0.12; 0.25], *p* = 0.4717), and anterior tear (β = 4.15, [−2.6; 10.91], *p* = 0.2277) were not associated with SST.

**Figure 1:**
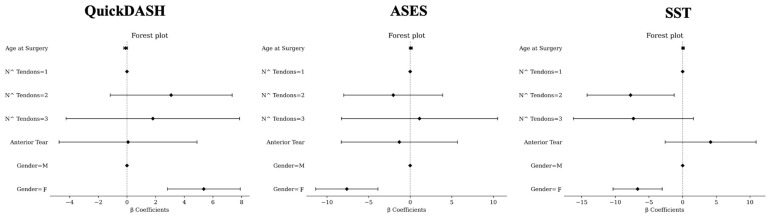
Multivariate analysis for QuickDASH (**left**), ASES (**middle**), and SST (**right**). M, male; F, female. For number (N^) of tendons, 1 tendon involved was considered as reference. For sex, male gender was considered as reference.

## 4. Discussion

The primary finding of this study is the long-term efficacy of single-row arthroscopic rotator cuff repair with metallic anchors, evidenced by excellent PROM scores over a minimum follow-up of 12 years. This technique yielded high levels of patient satisfaction, with a significant majority achieving functional outcomes classified as excellent or good across all PROMs. Median QuickDASH scores demonstrated excellent outcomes for most patients, suggesting minimal residual functional limitations, with excellent upper arm function and highlighting the durability of this approach.

Female patients, however, reported slightly lower functional scores on QuickDASH, ASES, and SST compared to male counterparts. This gender difference in PROMs after arthroscopic rotator cuff repair was previously reported in the literature at shorter follow-up [10] and was confirmed in our study even at long-term follow-up. Despite this variation, female patients reported satisfaction levels comparable to those of male patients (96% vs. 97%), underscoring the importance of subjective satisfaction even when functional scores are marginally lower. These results are probably explained by the fact that the differences are under the minimal clinical important difference for the considered PROMs [8,11,12] and their influence on overall patient satisfaction are limited. Moreover, factors beyond objective measurements, potentially including psychosocial and pain perception differences, may influence reported outcomes.

Age, the number of tendons repaired, or the anterior extension of the tear showed no significant impact on long-term satisfaction (except for SST after multivariate analysis, comparing patients with 2 tendons vs. 1 tendon repaired), reinforcing the single-row technique’s robustness across different age groups and tear complexities. This finding is consistent with the previous literature, suggesting that patient-centered outcomes such as satisfaction are largely independent of age and the extent of tendon involvement [13]. Thus, the single-row technique, which is both time-efficient and resource-conscious, can reliably deliver high satisfaction rates irrespective of patient demographics.

The literature shows a retear rate as high as 21% at 12–24 month follow-up [14]. Nevertheless, these findings prompt a reconsideration of the necessity for more complex, multi-anchor techniques, which have been advocated to reduce retear rates but may not improve patient-centered outcomes. As such, the single-row approach presents a compelling option that is cost-effective and clinically effective, achieving long-term patient satisfaction with less surgical complexity and resource use [15].

### Strengths and Limitations

This study’s strengths include a large patient cohort and an extensive minimum follow-up, ensuring comprehensive long-term data. However, the absence of preoperative PROMs limits insight into the degree of improvement from the baseline, and the lack of periodic follow-up precludes analysis of score stability over time. The 62.8% follow-up rate may introduce response bias, although prior studies indicate that response rates above 50% sufficiently mitigate bias risk [16]. The monocentric nature of the study may introduce geographical, cultural and operator biases. Finally, the impact of other associated procedures, such as bicep tenotomy/tenodesis or subacromial decompression, were not considered in the analysis.

## 5. Conclusions

This study demonstrates that single-row arthroscopic rotator cuff complete repair with metallic anchors is associated with excellent long-term patient satisfaction and PROMs, even after 12 years. The results highlight that the female gender is a predictor of slightly lower functional scores, although satisfaction remains consistently high across genders. Neither age nor the number of tendons repaired significantly influenced long-term satisfaction, underscoring the reliability of this cost-effective and time-efficient approach. These findings emphasize the durability of the single-row technique in meeting patient expectations and preserving function over the long term, despite ongoing advancements in more complex surgical methods.

## Figures and Tables

**Table 1:** Demographic characteristics of the population.

Parameters	n
Total Number of Patients	628
Age: mean ± SD (range) [years] <50 years old 50–59 60–69 ≥70	57.6 ± 9.6 (16.1 to 78.4)
	125
	234
	215
	54
Sex: Males (%)	332 (53%)
Lesion pattern * N^ tendons • Single tendon ○ Supraspinatus ○ Subscapularis ○ Infraspinatus • 2 tendons • >2 tendons	433 (69%) 419 12 2 134 (21%) 61 (10%)
Presence of anterior tear (Subscapularis involvement)	149 (24%)
Follow-up: mean ± SD (range) [years]	12.9 ± 1.3 (12–15)

* All the tendon tears were repaired at the end of surgery.

## Data Availability

Data where obtained through “WHospital medical archive” in Humanitas research hospital and are unavailable due to privacy restrictions.
